# The DPF Domain As a Unique Structural Unit Participating in Transcriptional Activation, Cell Differentiation, and Malignant Transformation

**DOI:** 10.32607/actanaturae.11092

**Published:** 2020

**Authors:** N. V. Soshnikova, A. A. Sheynov, Eu. V. Tatarskiy, S. G. Georgieva

**Affiliations:** Institute of Gene Biology Russian Academy of Sciences, Moscow, 119334 Russia; Engelhardt Institute of Molecular Biology, Russian Academy of Sciences, Moscow, 119991 Russia

**Keywords:** DPF domains, tandem PHD, MOZ and MORF histone acetyltransferases, DPF1, DPF2, DPF3, PHF10, BAF, PBAF

## Abstract

The DPF (double PHD finger) domain consists of two PHD fingers organized in tandem. The two PHD-finger domains within a DPF form a single structure that interacts with the modification of the N-terminal histone fragment in a way different from that for single PHD fingers. Several histone modifications interacting with the DPF domain have already been identified. They include acetylation of H3K14 and H3K9, as well as crotonylation of H3K14. These modifications are found predominantly in transcriptionally active chromatin. Proteins containing DPF belong to two classes of protein complexes, which are the transcriptional coactivators involved in the regulation of the chromatin structure. These are the histone acetyltransferase complex belonging to the MYST family and the SWI/SNF chromatin-remodeling complex. The DPF domain is responsible for the specificity of the interactions between these complexes and chromatin. Proteins containing DPF play a crucial role in the activation of the transcription of a number of genes expressed during the development of an organism. These genes are important in the differentiation and malignant transformation of mammalian cells.

## INTRODUCTION

The DPF (double PHD finger) domain belongs to the group of PHD (plant homeodomains) fingers, widely found in mammals. In humans, there are about two hundred PHD-containing proteins. The PHD domains have a zinc-finger (Zn-finger) structure. They consist of two antiparallel beta sheets and a C-terminal alpha helix. These structural elements are stabilized by two zinc ions coordinated by the Cys4-His-Cys3 motif [1, 2]. Although the primary structure of PHD fingers is quite diverse, their secondary structure, described for the first time in 2000, is highly conserved [3].

PHD fingers are mainly found in proteins that interact with the N-terminal fragments of histones; they regulate gene expression [4]. PHD fingers bind to the N-terminal regions of histone H3, which can exist in various modifications [5, 6].

Some proteins contain only one PHD-finger domain, while others may contain several, consecutive PHD fingers that function either independently of each other or in concert.

The DPF domain is a tandem of PHD fingers with a face-to-back orientation. Two domains form a single structure interacting with the N-terminal fragments of histones in a manner different from that for independent PHD-finger domains. Our review focuses on proteins containing the DPF domains, their organization, molecular mechanisms of recognition of histone tails, the impact on gene expression, as well as their role in mammalian development and oncogenesis.

## PROTEINS AND COMPLEXES CONTAINING THE DPF DOMAIN

Proteins containing the DPF domain mostly are the subunits of large protein complexes that determine and change the epigenetic status of chromatin [6]. The specific function of these complexes is ensured by precise recognition of the epigenetic modifications of chromatin, most of which are the modified N-terminal fragments of histones. Many subunits of the complexes contain different domains that interact with histones. For instance, these domains include the bromodomain (TAF1 and BAF180 proteins), chromodomain (CHD1 protein), Tudor domain (Uhrf1 protein), and their combinations. Each of the domains can recognize a specific modification of the N-terminal histone sequence. Acting together in a combinatorial manner, they increase the number of chromatin marks that are recognized by the full complex.

**Fig. 1:**
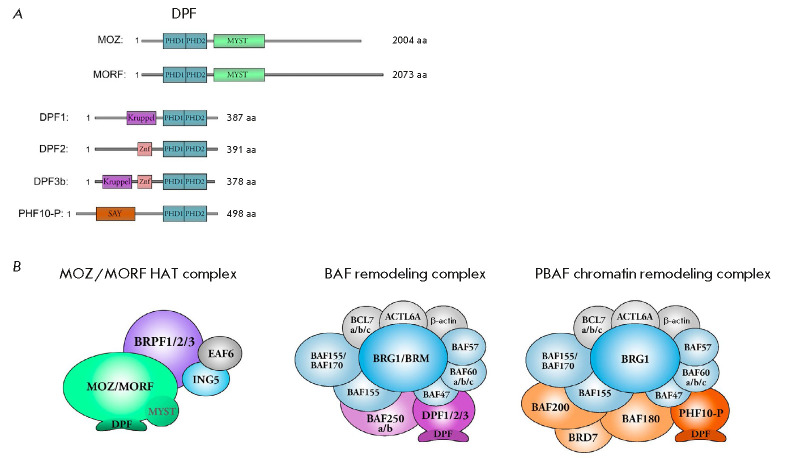
Proteins and complexes containing the DPF domains. (*A*) – Domain organization of the MOZ, MORF, DPF1, DPF2, DPF3b, and PHF10-P proteins. The DPF domains are shown with blue boxes. (*B) *– Schematic representation of the complexes containing the corresponding proteins with the DPF domains: histone acetyltransferase MYST-family HAT complex; BAF and PBAF chromatin remodeling complexes

The DPF domain is found in two groups of proteins. The first group includes the histone lysine acetyltransferases MOZ and MORF, while the other one is represented by proteins of the SWI/SNF chromatin remodeling complex (*Fig. 1A*). Acetyltransferases MOZ (also known as MYST3/KAT6a) and MORF (MYST4/KAT6b) are paralogs. They are alternatively contained within the MYST-family histone acetyltransferase (HAT) complex, which acetylates the N-termini of histones [7, 8] (*Fig. 1B*). The HAT complex is a transcriptional coactivator that resides in open, actively transcribed chromatin. MORF and MOZ contain the MYST domain, which acetylates the lysine residues in the N-terminal sequences of histone H3 (H3K9, H3K14ac, and H3K23). MYST-family HAT is responsible for the hyperacetylation of chromatin regions, which promotes activation of the respective genes [8, 9, 10].

Another group of proteins containing DPF is found in the SWI/SNF chromatin remodeling complex (its BAF and PBAF subfamilies) (*Fig. 1B*). This group includes the DPF1 (also known as BAF45b), DPF2 (REQ, or BAF45d), and DPF3 (BAF45c) (which are also called d4-family proteins), as well as PHF10 (BAF45a) proteins (*Fig. 1A*). The SWI/SNF complex is involved in the regulation of gene transcription, repair, and replication. Due to the ATPase activity of the major subunit of BRG1 and its homolog BRM, the complexes displace nucleosomes along the DNA strand or transfer the nucleosome to another DNA strand, remove H2A and H2B, as well as replace the canonical histone with its variant [11].

As mentioned above, the SWI/SNF family involves two types of complexes: BAF and PBAF (*Fig. 1B*). They share identical proteins in their core parts, which displace nucleosomes along the DNA strand. However, these complexes differ in the proteins within specific modules that are responsible for the interactions with chromatin. DPF proteins are components of the specific modules of the BAF and PBAF complexes and are involved in determining the specificity of complex binding to chromatin. The DPF domains present in these complexes are also involved in performing this function.

## THE STRUCTURAL FOUNDATIONS FOR HISTONE RECOGNITION AND THE SPATIAL ARCHITECTURE OF THE DPF DOMAINS

**Fig. 2:**
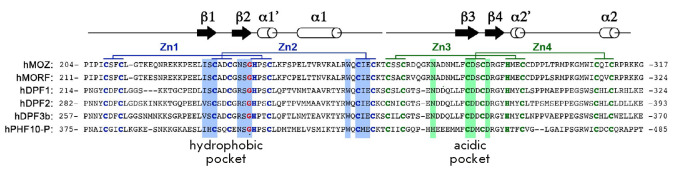
Alignment of the amino acid sequences of the DPF motifs of the MOZ, MORF, DPF1, DPF2, DPF3b, and PHF10-P human proteins. Schematic representation of the secondary structures of PHD1 and PHD2 is shown above the sequences. Cysteine and histidine residues coordinating Zn ions in PHD1 and PHD2 are indicated in blue and green, respectively. Homologous amino acids in PHD1 that form a hydrophobic pocket and bind H3K14ac/cr are highlighted in blue. Homologous amino acids that form an acidic pocket and bind to the first to fourth N-terminal amino acids of histone H3 are highlighted in green

The DPF domains of the MOZ, MORF, DPF1, DPF2, DPF3b, and PHF10 proteins are highly homologous; their secondary structures are formed by the same key amino acids (*Fig. 2*). Therefore, the data obtained for the DPF domains of each of these proteins are likely to be true for the DPF domains of other proteins belonging to this group.

The structure of each of the two PHD domains of the MOZ, MORF, DPF1, DPF2, DPF3b, and PHF10 proteins is typical of zinc finger domains. It consists of two antiparallel beta sheets, followed by an alpha helix, which are coordinated by two zinc atoms via the Cys4-His-Cys3 motif (*Fig. 2*). However, as shown for the MOZ protein, two PHD fingers are associated with each other in a face-to-back manner through the interaction between E247 and R251 in the alpha helix of the first PHD finger, as well as interaction between S283 and R286 in the third and fourth beta sheets of the second PHD finger.

The carboxyl and carbonyl groups of E247 form two hydrogen bonds with two water molecules, which interact with the carboxyl and carbonyl hydrogen atoms in S283. R251 interacts with the nitrogen atom in the R286 side chain in a similar way. Thus, these polar interactions localize the two PHD fingers, which form a unique globular structure [12]. The DPF of the DPF2, DPF3b, and MORF proteins also form a similar integral structural unit [13, 14].

## THE DPF DOMAINS INTERACT WITH ACYLATED H3K14 AND H3K9

**Fig. 3:**
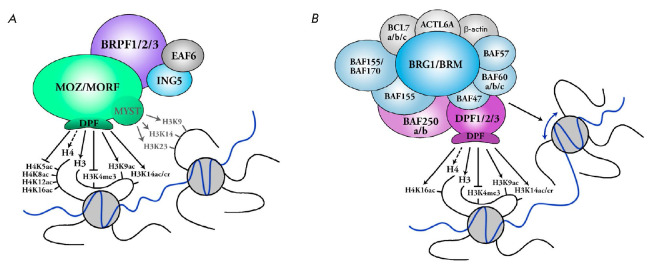
MOZ/MORF or DPF1-3 protein. The interaction between the DPF domains and histone modifications (black arrows), as well as the histone acetyltransferase activity of the MYST complex (gray arrows) (*A*) and the chromatin remodeling activity of the BAF complex (blue arrow) (*B*), is shown

The DPF modules of the MOZ, MORF, DPF2, and DPF3b proteins interact with unmodified N-terminal fragments of histone H3. Acetylation of H3K14 and H3K9 increases the binding constant threefold [12-15]. Methylation of H3K9me3 does not affect binding, while methylation of H3K4me3 severely inhibits DPF binding to histones (*Fig. 3*) [16]. The DPF domain of these proteins also weakly interacts with the N-terminus of histone H4. Acetylation of the H4K5, H4K8, H4K12, and H4K16 lysine residues abolishes the interaction between histone H4 and the DPF domains of MOZ and MORF (*Fig. 3*) [16].

A short time later, it was shown that the DPF domains of the MOZ and DPF2 proteins can interact with crotonylated Lys14 in histone H3 (H3K14cr) [17]. The crotonyl group has a more hydrophobic side chain residue and forms a planar spatial structure. The DPF domains of acetyltransferase MORF interact with other acyl groups, such as the butyrylated (H3K14bu), succinylated (H3K14su), and 2-hydroxyisobutyrylated H3K14 (H3K14hib) lysine residues. These groups also have longer hydrophobic side chains compared to those in acetylated modifications [18].

The molecular mechanism of interaction between DPF and various modifications of histone H3 has been studied using crystal structures of the DPF domains with either unmodified histone tails or various modifications (H3K14ac/cr). Both PHD domains form a single structural unit and bind the N-terminal fragment of histone H3 to one of the following modifications: H3K14ac, cr, or bu [12, 17, 18]. Among these, the crotonyl group is the preferential modification for binding the DPF domains in MORF. MORF DPF binds to H3K14cr three times more strongly than to H3K14ac [19]. It has been shown quite recently that a small DPF region in MORF (within the R306–K309 residues) interacts with DNA. These interactions are determined by the H3K14cr modification and enhance the binding of MORF to the nucleosome [19].

## THE MECHANISM THROUGH WHICH THE DPF DOMAIN RECOGNIZES POST-TRANSLATIONAL MODIFICATIONS OF HISTONES

The first PHD domain of the MOZ, MORF, DPF2, and DPF3b proteins is a unique zinc-finger type domain. It contains a hydrophobic pocket for binding acylated lysine (*Fig. 2*). Although the acylated H3K14 occupies the same pocket within the PHD1 domain of MOZ and DPF3b, different amino acids are involved in the interaction between H3K14 and DPF in the MOZ and DPF3b proteins [16]. However, the hydrophobic pocket within the beta-2 sheet in the first PHD finger is a common structural feature required for the binding of the H3K14ac, H3K14cr, and H3K14bu modifications [17, 18]. In the MOZ and MORF proteins, the hydrophobic pocket is formed by the amino-acid residues N235–G237 of the beta-2 sheet, I228–C230 of the beta-1 sheet, and amino-acid residues S210 (S217), F211 (F218), L242 (L249), W257 (W264), C259 (C266), I260 (I267), and E261, which coordinate the zinc ion (*Fig. 2*).

G237 is the most important amino-acid residue for the formation of this pocket, which recognizes acetylated and crotonylated groups (*Fig. 2*). This glycine residue is present in the DPF domains of the MOZ, MORF, DPF1, DPF2, DPF3b, and PHF10 proteins, indicating that the DPF domains in all these proteins can interact with acetylated, crotonylated, and butyrylated H3K4 (H3K4ac/cr/bu) (*Fig. 2*) [12, 17]. The F211 (F218) residue is responsible for the differences in the interaction between H3K14cr and H3K14bu as it can form π−π interactions between the aromatic ring of phenylalanine and the C=C double bond of the crotonyl group [19].

The second PHD domain of the MOZ and MORF proteins is organized in such a way that the first four H3K14ac/cr/bu residues of the peptide bind to the “acidic” pocket within the beta-1 sheet of this PHD2 domain. It is important that the amino group residues R2 and K4 are not methylated. The side chain of the H3R2 peptide is kept in its place by five hydrogen bonds of the DPF module of the MOZ protein with the C281, D282, and D285 residues. Meanwhile, the E261 and N274 residues form hydrogen bonds with the amino group in H3K4. As a result of such spatial restriction of the R2 and K4 residues, any methylation breaks the bond between DPF and H3, while binding of acetylated lysine residues preferentially occurs [12, 14, 17]. There is increasing experimental evidence that the second PHD domain of d4-family proteins is organized according to the same principle and is unable to recognize methylated H3K4 [5].

These data have been confirmed by *in vivo *experiments: it was shown that MOZ is associated with H3K14ac-rich chromatin and does not bind to H3K4me3-marked chromatin [16]. Crotonylated H3K14cr marks were found in the same genes (*Hox9A*, *Hox7*, and *Hox5A*) to which histone acetyltransferase MOZ binds [17]. There is still no explanation for the presence of two mutually exclusive modifications, H3K14ac and H3K14cr, in the same genes. Apparently, the presence of a H3K14 modification strongly depends on the metabolic pathways active in the cell, since the percentage of crotonylated or butyrylated histones is directly related to the amount of the respective acyl- CoA available for involvement in the metabolic pathways [17, 20, 21]. Therefore, HATs can switch between substrates to change the types of acylation profile of modified histones.

## THE DPF DOMAIN IN TRANSCRIPTIONAL REGULATION

As mentioned above, the DPF domains bind to the acylated (acetylated, crotonylated, and butyrylated) tail of histone H3 and act as the so-called readers: i.e., the proteins recognizing these histone modifications. The H3K14ac and H3K9ac modifications interacting with the DPF are characteristic of transcriptionally active chromatin. Histones highly enriched in these modifications are located in gene promoters and enhancers [22, 23]. Crotonylation of histone H3 (H3K14cr) is also found in transcriptionally active chromatin.

The mechanism of regulation of transcription and the epigenetic state of chromatin by HAT complexes involving MOZ and MORF has been shown for the *HOX9A *gene. The complexes are recruited to chromatin after their interaction with certain transcriptional activators (e.g., RUNX and P53) [24, 25] or due to the interaction of other subunits with various modifications [26, 27, 28]. The DPF domains of MOZ and MORF promote the localization of the complex in the H3K14ac-containing regions [12, 14], while the DNA-binding DPF motifs can stabilize these interactions with the nucleosome [19]. H3K14 acetylation is predominantly performed by histone acetyltransferase HBO1, which also contains the MYST domain [29]. However, the MOZ/MORF proteins can also induce this modification [30]. Acetylation of H3K23 and H3K9 is mainly carried out by the MYST domain of the MORF protein [31, 32] and can occur either in the same nucleosome or in an adjacent one [19]. Acetylation of the adjacent nucleosome contributes to changes in the complex localization and its transfer to the adjacent nucleosome. A similar mechanism drives the spread of histone marks between nucleosomes, thus resulting in the formation of hyperacetylated chromatin domains. The recruitment of the HAT complex to some *HOX *genes (*HoxA9*, *HoxA7*, *HoxA5*, and *HoxD13*) and formation of hyperacetylated domains in the promoters of these genes enhances their expression [12, 16, 17, 21]. Later, a genome-wide analysis of the ENCODE database revealed that the H3K23ac and H3K14ac modifications are co-localized and that the promoter regions of highly transcribed genes are rich in these modifications in IMR90, hESC, and HMEL cell lines [19, 33].

The SWI/SNF family complexes, which comprise another group of DPF-containing proteins, are more varied in terms of their protein composition than MYST acetyltransferase complexes. Combinations of different subunits are responsible for the specific composition of the complex, where the unique pattern of the domains binding DNA or histones in these complexes localizes the remodeling complex within specific chromatin regions. The BAF and PBAF complexes are recruited to certain loci by transcriptional activators and are involved in nucleosome remodeling: they shift the nucleosomes along the DNA strands and remove histones H2A and H2B [34]. Remodeling complexes abundantly occur in enhancers, which also supports the fact that the remodeling complexes are involved in transcriptional activation [35, 36].

The DPF1, DPF2, DPF3b, and PHF10 proteins also act as transcriptional coactivators. DPF3b and DPF3a bind to the NF-kB activator and are recruited together, within the BAF complex, to the *IL-6 *promoter in response to TNF-α stimulation [37]. The PHF10 protein in the PBAF complex coactivates the transcription of various genes [38, 39]. Direct evidence has been obtained that the DPF domain of the PHF10 protein is required for transcriptional activation, since a protein lacking DPF cannot activate transcription [38, 40]. Interestingly, the PHF10 isoform lacking the DPF domain is also found in cells [39]. The isoform containing the DPF domain is involved in transcriptional activation, while the isoform lacking DPF is needed to maintain a steady-state transcriptional level after activation [40]. Therefore, the DPF domain in the PHF10 protein is a potent transcription coactivator.

## DPF IN THE REGULATION OF CELL DIVISION AND DIFFERENTIATION. ITS ROLE IN TISSUE DEVELOPMENT IN MAMMALS

Since their discovery, MOZ and MORF have been associated with the regulation of cell proliferation. The interaction of MOZ with PML and P53 in MCF7 breast cancer cells and mouse embryonic fibroblasts (MEFs) was shown to result in acetylation of P53, followed by activation of p21 expression. The p21 protein is a cell cycle inhibitor that suppresses the cyclin E/CDK2 complex. The cyclin E/CDK2 complex phosphorylates a number of factors promoting gene activation in the G1/S checkpoint of the cell cycle. When unable to trigger gene expression for the G1/S transition, cells exit the cell cycle and stop dividing. Therefore, MOZ and MORF inhibit proliferation and implement the subsequent scenario of cell transition to senescence [25, 41]. Meanwhile, MOZ maintains the expression level for some genes coding for senescence inhibition in the INK4/ARF locus via the H3K9ac modification [42, 43]. As described above, MOZ and MORF regulate the expression of many *HOX *genes responsible for organism development and differentiation. This partially occurs through their interaction with factor BMI1, which has been demonstrated at the genetic level [44].

MOZ plays an important role in maintaining the pool of embryonic hematopoietic stem cells in mammals. Knockout mice died at the E14.5 embryonic stage and exhibited manifestations of liver and hematopoietic pathologies [45]. The *MOZ *gene is also required for the normal development of blood B cells and progression of c-MYC-induced lymphoma. MOZ interacts with AML1 and PU.1, two important hematopoietic factors, and acts as their coactivator by ensuring accurate expression of the respective genes [46, 47].

The recent genome-wide studies of patients with congenital abnormalities (severe speech disorders, hypotension, and facial dysmorphism) revealed mutations in the *MOZ *gene [48]. MORF is actively involved in the development of neural and bone tissue. Mice with a minimal amount of MORF RNA (~10%) had dwarfism, craniofacial disorders, and cerebral defects [49]. MORF plays a crucial role in the regulation of neuronal stem cells; it is required to maintain neurogenesis in adult mice [50]. Thus, although MOZ and MORF are interchangeable *in vitro*, they play different roles *in vivo*: MOZ is important for hematopoiesis, while MORF is involved in neurogenesis and osteogenesis.

The DPF proteins within the SWI/SNF complexes are required for neurogenesis in mammals. DPF3b, a component of the BAF chromatin remodeling complex, plays a crucial role in the differentiation of muscle and cardiac tissues [51]. PHF10 is expressed in nerve cell precursors from the early embryonic stages. Its expression reduces after birth. PHF10 can maintain the proliferation of neuronal progenitor cells. As a component of the PBAF complex, it binds to the promoters of the signaling pathway genes regulating neuronal proliferation and differentiation: the Notch, SHH, and various transcription factors. Other DPFs (DPF1, 2, and 3) begin to be expressed in the mouse brain at later stages, starting from E13, and are unable to maintain proliferation of neuronal cells [38]. DPF1 is presumably important for the functioning of adult neurons, since it is expressed tissue-specifically only in the brain of adult mammals. DPF2 is also involved in the development and function of the nervous system. Single-nucleotide substitutions affecting the sequence of DPF domains and disrupting the binding of DPF2 to acetylated H3 were found in patients with the Coffin-Siris syndrome. This disease manifests itself as cognitive dysfunction and intellectual impairment of varied severity, coarse facial features, and brain abnormalities such as hypoplasia and agenesis of the corpus callosum [52].

The DPF2 (the BAF complex) and PHF10 (the PBAF complex) proteins are expressed in hematopoietic progenitor cells of E14.5 mouse embryos and regulate their differentiation [53]. DPF2 inhibits myeloid differentiation of hematopoietic progenitor cells. Its DPF domain is responsible for the recruitment of DPF2 and the entire BAF complex to specific acetylated chromatin loci: the binding sites of the transcription factor RUNX1. This factor promotes the myeloid differentiation of progenitors. DPF2 knockdown in CD34+ cells reduces the expression of the genes involved in mitosis and cell cycle regulation; it also disrupts the transcription of the genes involved in differentiation [15].

The homozygous PHF10 knockout causes death of mouse embryos (E19), while conditional knockout in the hematopoietic cells of an adult mouse causes significant depletion of myeloid precursors (granulocytes). An analysis of RNA isolated from these cells showed that PHF10 significantly affects the expression of cell cycle genes [53]. A study performed using a model HL-60 cell line, which is capable of myeloid differentiation, and terminally differentiated human neutrophils showed that PHF10 isoforms containing the DPF domain play a crucial role in the maintenance of proliferating myeloid progenitors. These isoforms are also required for the activation of specific myeloid genes whose expression is activated during differentiation. In mature neutrophils, transcription of specific genes is maintained by PHF10 isoforms lacking DPF [40].

## THE ROLE OF PROTEINS CONTAINING DPF DOMAINS IN THE MALIGNANT TRANSFORMATION OF CELLS

Mutant proteins containing the DPF domain are often found in tumor cells. Abnormal expression of MOZ and MORF is often associated with different types of leukemia. Chromosomal regions where the *MOZ *and *MORF *genes are located undergo various translocations, giving rise to chimeric proteins [10]. Myeloid leukemia is accompanied by translocations between the *MOZ *and *CBP *genes [54]. Acute monocytic leukemia is associated with translocations between the *MOZ *and *P300 *genes [55]. Translocations between *MOZ *and *LEUTX *[56], as well as other genes [57], are observed in acute myeloid leukemia. *MORF *can also undergo translocation to form chimeric proteins. This translocation gives rise to the MORF-CBP chimeric protein, which is associated with acute myeloid leukemia [58, 59]. Chimeric proteins resulting from translocations carry DPF domains at their N-termini, leading to the recruitment of a new modifier, an activator or a regulator of the old chromatin environment that used to be occupied only by a MYST family acetyltransferase.

It has been found that MOZ is required to maintain the progression of lymphoma induced by the *MYC *oncogene [60]. The lack of this protein causes senescence of neural stem cells [43]. Increased MOZ expression promotes glioblastoma and breast cancer development [61, 62, 63].

D4 and PHF10 family proteins are rarely mutated in cancer cells [64, 65]. However, decreased DPF2 expression correlates with a poor survival prognosis in patients with glioma [66]. It was also shown that DPF2 maintains the proliferation of transformed MLL-AF9 myeloid progenitor cells. Upon DPF2 knockdown, the cells started to differentiate, exit the cell cycle, and undergo apoptosis [67].

No significant associations between changes in DPF1 expression and malignant cell transformation were found in cancer patients.

Decreased PHF10 expression in patients with renal cancer correlates with a higher chance of patient survival [64, 66], which may be related to the positive effect of the *c-MYC *oncogene on PHF10 expression [68].

Almost no DPF3 expression is observed in human myeloid precursors. However, due to the action of STAT5, its expression significantly increases in the granulocytes of patients with chronic lymphocytic leukemia. Apparently, this may cause transcriptional dysregulation and disease progression [69]. Decreased DPF3 expression is also associated with poor survival prognosis in breast cancer patients. Thus, reduced DPF3 expression was shown to activate the JAK2/ STAT3 signaling pathway and enhance the mobility of cancer cells [70].

## CONCLUSIONS

The implementation of different transcriptional pathways involves transcription factors (namely, activators and repressors), as well as various auxiliary complexes that change the chromatin structure. These complexes usually consist of a large number of subunits containing numerous diverse domains that bind DNA and specific chromatin marks, which are known as modified histone tails. Due to these domains, the complexes are positioned in a strictly defined chromatin region and further additionally modify it through their activity. The DPF domains form a unique structure that binds the histone H3 tail favoring the modified H3K14ac/cr. H3 histones with acetylated and crotonylated lysine residues mainly reside in either the promoter or enhancer regions of transcriptionally active chromatin. Therefore, they act as markers for recruiting the MYST-family HAT and BAF/PBAF complexes involving proteins containing the DPF domains. There are few proteins that contain DPF. However, these proteins have homologous DPF sequences and identical amino acids at the key positions which determine the binding to H3K14ac/cr. The MYST-family HAT and BAF/ PBAF complexes acetylate other histone tails and remodel (translocate) nucleosomes, respectively; i.e., they function as coactivators and contribute to additional transcriptional activation.

Thus, DPF domains perform the important function of binding chromatin, which leads to the activation of the transcription of the genes that play a crucial role in the development of an organism.
